# IgE-Dependent Food Sensitisation and Its Role in Clinical and Laboratory Presentation of Paediatric Inflammatory Bowel Disease

**DOI:** 10.3390/nu15081804

**Published:** 2023-04-07

**Authors:** Anna Buczyńska, Urszula Grzybowska-Chlebowczyk, Krzysztof Pawlicki

**Affiliations:** 1 Department of Pediatrics, Faculty of Medical Sciences in Katowice, Medical University of Silesia, 40-055 Katowice, Poland; 2 Department of Biophysics, Faculty of Medical Sciences in Katowice, Medical University of Silesia, 40-055 Katowice, Poland

**Keywords:** paediatric IBD, food allergy, IgE sensitisation, inflammatory bowel disease

## Abstract

The rising prevalence of inflammatory bowel disease (IBD) and food allergies and their partially overlapping mechanisms such as microbiome diversity reduction raise questions about the role of allergies in IBD. While data on their comorbidity are available, analysis of IgE-sensitization’s influence on the clinical presentation of IBD is lacking and is the aim of this study. Histories of 292 children with newly diagnosed IBD (173 cases of ulcerative colitis, 119 cases of Crohn’s disease) were analyzed. Disease age of onset, activity, location, behaviour, and anthropometric and laboratory parameters were tested for its dependence on the presence of chosen IgE sensitization markers. A.o. Chi2, OR and phi coefficient were assessed. In Crohn’s disease (CD), elevated total IgE (tIgE) correlated with weight loss, rectal bleeding, ASCA IgG positivity (φ = 0.19 for all) and negatively correlated with complicated disease behaviour (φ = −0.19). TIgE > 5 × reference range correlated with being underweight (φ = 0.2), ASCA IgG positivity (φ = 0.3), ASCA double (IgA and IgG) positivity (φ = 0.25) and elevated total IgG (φ = 0.18). The presence of specific IgEs (sIgE) correlated with extraintestinal manifestations of IBD (φ = 0.19): Egg white sIgE correlated with upper GI involvement (L4b) (φ = 0.26), severe growth impairment (φ = 0.23) and colonic mucosal eosinophilia (φ = 0.19). In ulcerative colitis, decreased IgA correlated with egg white sIgE (φ = 0.3), as well as the presence of any (φ = 0.25) or multiple sIgEs (φ = 0.2); the latter correlated also with elevated IgG (φ = 0.22), fever (φ = 0.18), abdominal pain (φ = 0.16) and being underweight (φ = 0.15). Cow’s milk sIgE correlated positively with growth impairment (φ = 0.15) and elevated IgG (φ = 0.17) and negatively with extensive colitis (φ = −0.15). Pancolitis correlated negatively with sIgE presence (φ = −0.15). In summary, single moderate and numerous weak but interesting relationships were observed.

## 1. Introduction

Inflammatory bowel diseases (IBDs) and their most common types—Crohn’s disease and ulcerative colitis—are immune-mediated chronic gastrointestinal diseases of complex, multifactorial background in genetically susceptible hosts. 

It is known that an adverse reaction to food is one of many mechanisms at the root of IBD. The mechanisms of abnormal immune system–gastrointestinal tract–food interactions are complex and include the modifying effect of the composition of the food on the gut microbiome and the gut microbiome on immunotolerance. Monosaccharides, saturated fatty acids, *n*-6 acids and food additives are well described as food components presenting direct, proinflammatory effects [1,2,3]. In the 1980s and 1990s, the mechanisms of IBD were explored, including the presence of mast cells and IgE in the patient’s bowel wall [4,5], histamine secretion [6], the role of eosinophilia and IgE as an inflammatory mediator in IBD aetiology [7,8]. Only rare and generally earlier papers did not support the involvement of the above-mentioned factors in the development of IBD [9,10]. Subsequent studies have demonstrated several mechanisms involved in the development of both IBD and allergic diseases, participating in the Th1 and Th17 pathways (particularly in Crohn’s disease) and the Th2 pathway (particularly in ulcerative colitis) [11,12]. A common pathogenetic pathway is also suggested by the demonstration of the involvement of IL10 signalling in stimulating tolerance to benign allergens; IL10 pathway impairment is a well-known mechanism not only in IBD but also in allergic rhinitis and asthma development [13]. 

In view of these observations, since the 2010s, the coexistence of allergic diseases and IBD has again been an object of interest for researchers. In various populations, attempts have been made to assess whether allergic diseases such as asthma, allergic rhinitis (AR), cow milk allergy (CMA), and atopic dermatitis occur more frequently in IBD than in the healthy population; usually, but not always, such a trend is shown [14]. The methodology often relied on a questionnaire survey to assess a diagnosed allergic disease or symptoms of such a disease in a patient with IBD [14,15,16]. Some authors have used tests dedicated to the assessment of sensitisation to allergens (sIgE assessment, skin prick tests) and even IgG against food proteins [17,18], although it is known that the latter is not a marker indicative of allergisation, but rather a normal reaction to contact with food proteins [19].

It should be emphasized that the available studies focus on the prevalence of allergic diseases in IBD, while an assessment of its influence on IBD history, course and complications is lacking. 

This study analysed whether the presence of laboratory markers of food sensitisation in children with newly diagnosed IBD determines a different course of bowel disease compared with children with Crohn’s disease or ulcerative colitis without these markers. An evaluation of total IgE concentration as a potential prognostic factor was also included in the analysis. Although total IgE is considered as not having a relevant role in the diagnosis of allergic diseases [20], it has been shown recently that sIgE/total IgE ratio analysis may be a more precise predictive marker of the result of oral food challenge than sIgE alone [21] and may play a minor role in the interpretation of food-specific IgE levels [22]. In addition, the change in concentrations of major class antibodies, such as the overproduction of IgA in Crohn’s disease [23,24] or the concentration of IgG and its subclasses, is also one of many immunological phenomena occurring in IBD and may have prognostic significance [25].

In conclusion, the aim of the present study was not only to assess the prevalence of IgE-dependent markers of food sensitisation in a population of children with IBD, but also the possible association between the presence of these markers or the presence of elevated total IgE and the IBD clinical presentation, disease severity, need for biological/surgical treatment and selected laboratory parameters including IgA and IgG levels.

## 2. Materials and Methods

We analysed the medical histories of 119 children with Crohn’s disease (CD) and 173 with ulcerative colitis (UC) who were diagnosed with IBD between 2009 (for CD) or 2005 (for UC) and 2019 at the Department of Paediatrics, Medical University of Silesia in Katowice, Poland. Data on children’s age, sex, nutritional status and growth and selected laboratory parameters such as levels of total IgA and IgG antibodies, total protein, albumin and haemoglobin were extracted from the first hospitalisation where the diagnosis of IBD was made. The phenotype of IBD at onset was also analysed according to the Paris classification [26,27]. For Crohn’s disease, patients were therefore classified as presenting with inflammatory (B1), structuring (B2) or penetrating disease behaviour (B3). In addition, disease locations such as L1—ileocecal disease; L2—Cohn’s colitis; and L3—both distal ileal and colonic involvement—were recorded. Upper GI tract involvement was also analyzed and described as L4a—proximal to the Treitz ligament; or L4b—distal to the Treitz ligament, reaching at maximum to 2/3 of the ileum. As known according to the Paris classification, L4a and L4b can coexist with the L1–L3 locations.

For ulcerative colitis, disease extension was recorded as E1—rectal disease; E2—distal to the splenic flexure; E3—distal to the hepatic flexure; and E4—pancolitis. Pancolitis presented with or without backwash ileitis or macroscopic rectal sparing. 

Data such as total IgE and specific IgE for selected food allergens (cow’s milk, egg white, egg yolk and wheat) and magnetic resonance enterography were included from up to 3 months after the first diagnostic hospitalization. Data on the occurrence of extraintestinal manifestations (EIM) or biological or surgical treatment in children (segmental bowel resection, ileostomy or colectomy were considered) were collected from the patient’s entire follow-up period in our Department, i.e., usually up to 18 years of age. In analysis, the parameter ‘surgical treatment’ in ulcerative colitis was omitted since it applied only to 2 children in UC (and, for comparison, to 12 children in the CD group). 

Using statistical tests, it was assessed whether the occurrence of abnormal IgE-dependent sensitisation parameters to foods correlated with the occurrence of specific clinical features of IBD among those listed above. The following statistical methods were used: Mann–U Whitney test for numerical variables (Pediatric Crohn’s Disease Activity Index—PCDAI, Pediatric Ulcerative Colitis Activity Index—PUCAI, or age) and for categorical variables, chi-square tests of independence, Fisher’s exact test when appropriate in small sample sizes (<5 people in any field of the contingency table), OR, RR (given with 95% confidence intervals in brackets) and measures of correlation were used. Of those, primarily phi (φ) coefficient, Pearson’s contingency coefficient C, Cramer’s V and Yule’s Q were assessed; however, in further analysis, phi was chosen as the single most universal one since some of analysed features were represented by small children subgroups. Therefore, Yule’s Q was for most tested parameters omitted, taking into account its strong dependence on sample size. Moreover phi (φ), representing values <−1,1>, presents both the strength and the direction of correlation. In our data, expressed in 2 × 2 contingency tables, phi, Cramer’s V and Pearson’s C were equal to the second decimal place. 

The grouping variables for the analysis of clinical features of IBD at diagnosis were the presence of cow’s milk, egg white sIgE ≥ 0.35 KIU/L, sIgE present in concentrations ≥ 0.35 or ≥0.7 KIU/L for more than 1 allergen in one patient (egg yolk and wheat were also assessed), the presence of elevated-for-age total IgE level and the presence of significant elevation of total IgE (considered to be a value at least five times the normal range). Therefore, in the analysis, a few study groups were compared with a few control groups: children presented with positive sIgE or tIgE elevation were compared with children negative for the analogous parameters. This study construction led to the analysis of 4 pairs of study–control groups in Crohn’s disease and 8 pairs in ulcerative colitis. 

sIgE testing was carried out using an immunoenzymatic chemiluminescence test. The presence of sIgE ≥ 0.35 KIU/L was chosen for testing although it is known that the probability of a diagnosis of allergy to a test antigen increases with increasing sIgE concentrations and depends also on the allergen assessed as well as the patient’s age and history [28], because the study sought to answer the research question of whether already the presence of food sensitisation markers at low concentrations, and not only the diagnosis of food allergy, correlates with the course of IBD. Total IgE, CRP, IgA and IgG levels were tested by immunoturbidimetry, and patients with high tIgE associated with ascaris lumbricoides or giardiasis were routinely excluded. Complete blood count was evaluated using fluorescein flow cytometry. ASCA and *p*-ANCA status was assessed by indirect immunofluorescence technique. Serum albumin levels and serum total protein levels were determined using quantitative colorimetry.

## 3. Results

### 3.1. Crohn’s Disease

**Figure 1:**
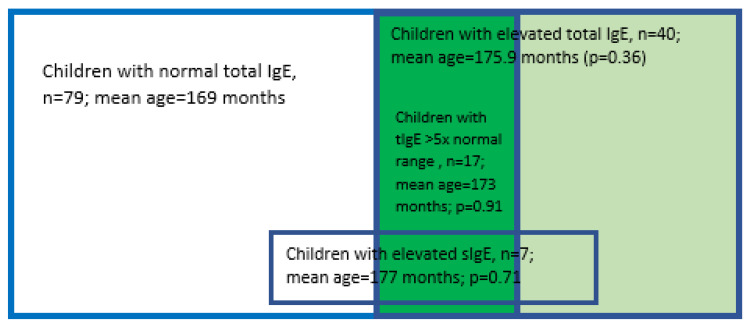
CD population structure.

The Crohn’s disease population consisted of 119 children, 75 boys (63%). Forty of these patients presented with elevated tIgE (in this group, 27 were boys, i.e., 67.5%) and in 17 amongst them, who represented 14% of the whole CD population, tIgE elevation exceeded the normal range more than 5-fold. Six of this subgroup were boys (35%). Seven children, representing 5.9% of the CD population, presented with one of the tested sIgEs (cow’s milk, egg white, egg yolk, wheat) ≥ 0.35 KIU/L (5, i.e., 71.4%, were boys). Of the sIgE positive patients, three presented with normal tIgE, one with tIgE elevated > 5 × reference range and two with lower tIgE elevation. The most frequent sIgE present was egg white sIgE, which was positive in six children (four, i.e., 67%, were boys). Before further analysis, in which we compared the distribution of numerous clinical and laboratory parameters in defined subpopulations, the mean age between subgroups was compared to exclude age as a confounding factor influencing differences in the analyzed parameters. No statistical differences in mean age between analyzed subgroup and the complementary part of the population were noted (Figure 1). 

In terms of clinical presentation, children with elevated total IgE (n = 40) at the diagnosis of Crohn’s disease more often presented with rectal bleeding (42.5 vs. 24.3%; chi2 = 4.1, *p* = 0.04; OR = 2.29 [1.01–5.17], *p* = 0.04; φ = 0.19), weight loss prior to diagnosis (62.5 vs. 42%; chi2 = 4.32, *p* = 0.037; OR = 2.27 [1.04–4.97], *p* = 0.04; φ = 0.19) and perianal disease (27.5 vs. 12.8%; chi2 = 3.9, *p* = 0.048; OR = 2.58 [0.99–6.74], *p* = 0.053; φ = 0.18). Total IgE elevation correlated marginally with a positive family history of IBD (12.5 vs. 3.8%; chi2 = 3.2, *p* = 0.07; OR = 3.6 [0.82–15.99], *p* = 0.09; φ = 0.16) and negatively correlated with complicated disease behaviour (22.5 vs. 41.8%; chi2 = 4.3, *p* = 0.037; OR = 0.4 [0.17–0.96], *p* = 0.04; φ = −0.19). Regarding laboratory parameters, children with elevated tIgE more frequently presented with IgG ASCA (65 vs. 44%; chi2 = 4.55, *p* = 0.03; OR = 2.33 [1.06–5.13], *p* = 0.03; φ = 0.19). 

Particularly high, i.e., exceeding 5 times the normal range, total IgE concentration affected 17 children, and these patients were more frequently underweight (58.8 vs. 30%; chi2 = 5.22, *p* = 0.02; OR = 3.2 [1.14–9.39], *p* = 0.03; phi = 0.2). This significantly elevated tIgE correlated also with the occurrence of extraintestinal manifestations (70 vs. 40.6%; chi2 = 5.3, *p* = 0.02; OR = 3.5 [1.15–10.7], *p* = 0.03; φ = 0.21). Regarding laboratory test results, this parameter correlated with antimicrobial antibody positivity. This tendency was strongest for ASCA IgG positivity (88 vs. 45%; chi2 = 10.86, *p* < 0.001; OR = 9.13 [1.9–42, *p* = 0.005]; phi = 0.3), was visible for ASCA double positivity (IgA and IgG: 64.7 vs. 30%; chi2 = 7.5, *p* = 0.006; OR = 4.2 [1.4–12.4], *p* = 0.009; φ = 0.25) and marginal for isolated ASCA IgA positivity (64.7 vs. 43.6%; chi2 = 2.6, *p* = 0.1; Fisher’s exact test, *p* = 0.08; OR = 2.38 [0.8–6.9], *p* = 0.1; φ = 0.15). Concomitantly, total serum IgG was more frequently elevated in children with very high total IgE (17.6 vs. 4.9%; chi2 = 3.8, *p* = 0.052; OR = 4.16 [0.9–19.3], *p* = 0.07; φ = 0.18). 

The presence of egg white sIgE was noted in six children who were more likely to present severe (greater than −2 SD relative to age norm) growth impairment (33 vs. 6%; chi2 = 6, *p* = 0.1; Fisher’s exact test, *p* = 0.07; OR = 7.6 [1.18–48.7], *p* = 0.03; φ = 0.23). Upper gastrointestinal involvement was also more frequent in this group, especially in location L4b (50 vs. 10.6%; chi2 = 8, *p* = 0.005; Fisher’s exact test, *p* = 0.03; OR = 8.4 [1.5–46.5], *p* = 0.015; φ = 0.26) and marginally in location L4a (66 vs. 34.5%; chi2 = 2.55, *p* = 0.1; Fisher’s exact test *p* = 0.1; OR = 3.8 [0.7–21.6], *p* = 0.1; φ = 0.15). In the egg white sIgE+ group, eosinophilic infiltration of the colonic mucosa was more frequently found on histopathological examination (33 vs. 8%; chi2 = 4.4, *p* = 0.04; Fisher’s exact test, *p* = 0.095; OR = 5.78 [0.9–35.98], *p* = 0.06; φ = 0.19). 

It was also tested whether the presence of any assessed sIgE was a factor associated with clinical features of Crohn’s disease—this was the case in seven children. Extraintestinal manifestations (EIM) were more common in this group (43 vs. 13.4%; chi2 = 4.46, *p* = 0.03; Fisher’s exact test, *p* = 0.07; OR = 4.85 [0.99–23.8], *p* = 0.052; φ = 0.19). Colonic eosinophilia was marginally more frequent (28.6 vs. 8%; chi2 = 3.3, *p* = 0.06; Fisher’s exact test, *p* = 0.13; OR = 4.6 [0.78–27], *p* = 0.09; φ = 0.17).

**Figure 2:**
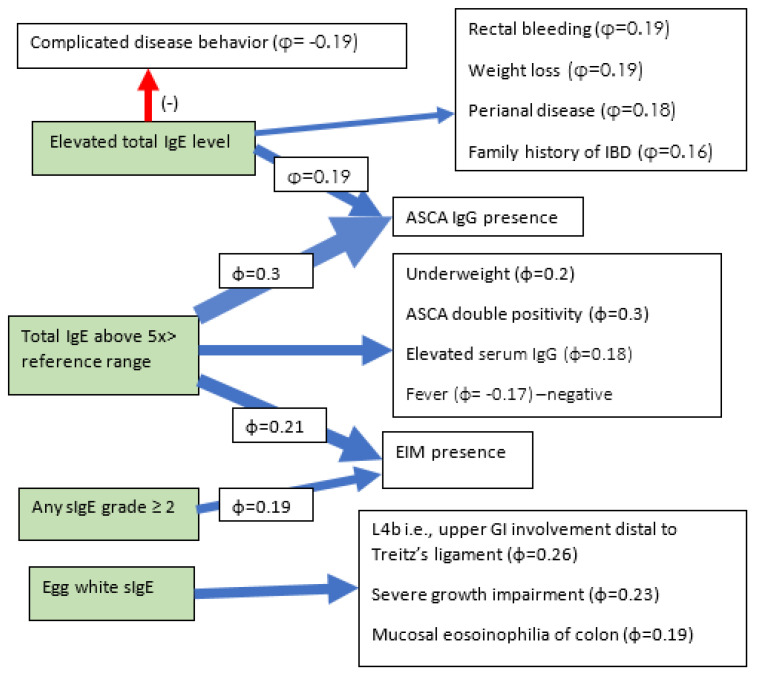
Correlations in CD.

**Figure 3:**
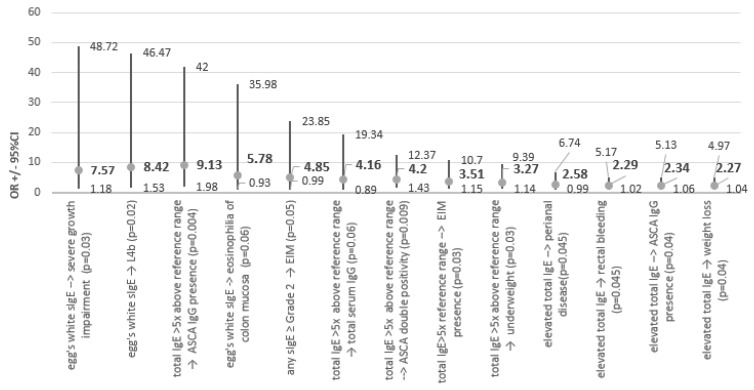
Crohn’s disease—OR.

Statistically significant correlations are shown in Figure 2 and Figure 3. 

Numerous other parameters were tested, such as the PCDAI level and the need for biologic or surgical treatment. No statistically significant results were noted. A summary of all parameters tested and whether or not they were correlating, is shown in Table S1 of Supplementary Materials. 

### 3.2. Ulcerative Colitis

The UC population consisted of 173 children, of which 94 were boys (54%). Sixty-one of these patients presented with elevated tIgE (31 boys; 50.8%). In 13 children (10 boys, i.e., 77%), who represented 7.5% of the whole UC population, tIgE elevation exceeded 5 times the normal range. Seventeen children, i.e., 9.8% of the UC population, presented with one of the tested sIgEs (cow’s milk, egg white, egg yolk, wheat) ≥ 0.35 KIU/L (nine, i.e., 53%, were boys). Ten children (seven boys; 70%) presented with sIgE for more than one allergen, and six of them (three boys) presented with multiple sIgE in at least 2two concentration classes (≥0.7 KU/L). Of 17 sIgE-positive patients, 12 presented with elevated tIgE, and amongst them, 3 had tIgE elevated > 5 × reference range. Concomitantly, of the 17 sIgE-positive patients, 13 had sIgE present for any of tested allergens ≥ 0.7 KIU/L. 

**Figure 4:**
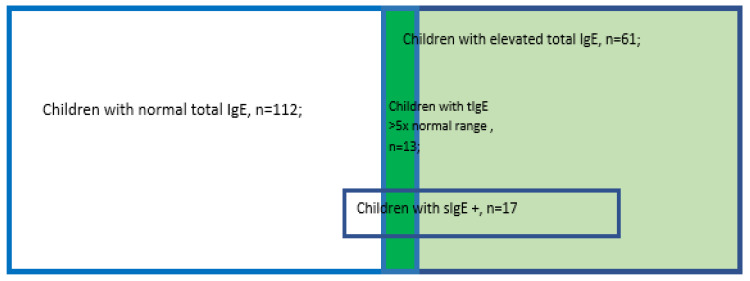
UC population structure.

The most frequently presented sIgE was egg white sIgE (10 children, of which 6, i.e., 60%, were boys) followed by cow’s milk protein sIgE noted in 6 children (2 boys; 33%). The structure of the group is presented in Figure 4.

The mean age between subgroups was compared to assess it as a potentially confounding factor influencing observed correlations. As shown in Table 1, children with cow’s milk, egg white sIgE or with the presence of any tested sIgE or with multiple sIgE positivity were, on average, 2.5–4.9 years younger than the rest of the UC population. Therefore, before reporting results regarding these grouping variables, it was assessed whether the tested parameters which had been shown to be significantly correlated or borderline with sIgE, also correlated with age. 

Regarding UC activity assessed using the Paediatric Ulcerative Colitis Activity Index (PUCAI), no significant differences between study and control subgroups were noted.

Children with abnormal total IgE (n = 61) were more likely to complain of abdominal pain than others at diagnosis of UC (56.6 vs. 40.9%; chi2 = 3.88, *p* = 0.049; OR = 1.9 [1–3.57], *p* = 0.05; φ = 0.15). Regarding laboratory test results hyperproteinaemia was marginally less common (8 vs. 17.9%; chi2 = 2.98, *p* = 0.08; OR = 0.41 [0.15–1.16], *p* = 0.09, 0.13; φ = −0.13). Histopathological examination of duodenal biopsies showed that eosinophilic infiltrates were also less frequent (11.5 vs. 23.2%; chi2 = 3.53, *p* = 0.06; OR = 0.43 [0.17–1.06], *p* = 0.06; φ = −0.14). 

Children with total IgE levels exceeding the age reference range by at least five times (13) were, compared with the others, more likely to present with weight loss before diagnosis (69 vs. 30%; chi2 = 8.16, *p* = 0.004; OR = 5.16 [5.16–17.56], *p* = 0.008; φ = 0.22). These patients also had a marginally higher incidence of extensive colitis (E3 or E4, according to the Paris classification) at the diagnosis of the disease (77 vs. 54%; chi2 = 2.48, *p* = 0.12; OR = 2.8 [0.74–10.55], *p* = 0.13; φ = 0.12). 

Extensive colitis was, at the same time, less frequent in children with cow’s milk sIgE present, which was found in a total of six children. E3 or E4 involvement affected 20% of children with cow’s milk sIgE compared with 57.5% in the group with this test negative (Fisher’s exact test, *p* = 0.06; OR = 0.15 [0.02–1.29], *p* = 0.08; φ = −0.15). Concomitantly, the presence of cow’s milk sIgE was marginally associated with a higher incidence of growth impairment at diagnosis of UC (50 vs. 17.7%; Fisher’s exact test, *p* = 0.08; OR = 4.66 [0.89–24.23], *p* = 0.07; φ = 0.15). And increase in total IgG levels was also more frequent in this group (50 vs. 15.6%, Fisher’s exact test, *p* = 0.06, OR = 5.42 [1.04–28.35], *p* = 0.045; φ = 0.17). Other than that, only statistical trends were noted, among them a more frequently recorded weight loss before diagnosis (66.7 vs. 32.1%; Fisher’s exact test, *p* = 0.09; OR = 4.23 [0.75–1.63], *p* = 0.1; φ = 0.13) and increased incidence of anaemia 83 vs. 47%, Fisher’s exact test, *p* = 0.09; OR = 5.57 [0.64–48.7], *p* = 0.012; φ = 0.13). 

Egg white sIgE positivity (10 children) was associated with IgA deficiency (30 vs. 3%; Fisher’s exact test, *p* = 0.007; OR = 12.5 [2.68–68.38], *p* = 0.002; φ = 0.3). At the same time, 13 children with any sIgE ≥ 0.7 KIU/L were marginally less likely to have WHO-grade moderate/severe anaemia (15.4 vs. 37.8%; Fisher’s exact test, *p* = 0.08; OR = 0.29 [0.06–1.34], *p* = 0.11; φ = −0.13) and pancolitis (15.4 vs. 42.5%; Fisher’s exact test, *p* = 0.047; OR = 0.25 [0.05–1.15], *p* = 0.07, φ = −0.15). Interestingly, in a study group extended to the presence of any sIgE ≥ 0.35 (17 children), only correlation with IgA deficiency remained visible—it concerned 23.5% of such a study group vs. 2.6% of the corresponding control group (Fisher’s exact test, *p* = 0.004; OR = 11.7 [2.62–52.25], *p* = 0.0013; φ = 0.3). Moderate/severe anaemia concerned 29% of such children vs. 38% of the control group (chi2 = 0.46, *p* = 0.49), and pancolitis (23.5 vs. 42%; Fisher’s exact test, *p* = 0.2).

Children with sIgE ≥ 0.7 KIU/L for more than one food allergen were analysed separately—there were seven such children. Abdominal pain was more common in this group compared to the rest of the patients (85.7 vs. 44.8%, Fisher’s exact test, *p* = 0.04; OR = 7.4 [0.87–62.83], *p* = 0.07; φ = 0.16) as well as fever (28.6 vs. 7.2%; Fisher’s exact test, *p* = 0.08; OR = 6.42 [1.06–38.7], *p* = 0.04; φ = 0.18). These children were more likely to be IgA deficient (28.6 vs. 3.6%; Fisher’s exact test, *p* = 0.04; OR = 10.67 [1.7–66.57], *p* = 0.01; φ = 0.23) and more frequently presented with total IgG elevation for their age (57.1 vs. 15%; Fisher’s exact test, *p* = 0.02; OR = 7.52 [1.59–35.65], *p* = 0.01; φ = 0.22). At the same time, some of these tendencies remained true for an extended group of children with multiple sIgEs already exceeding 0.35 KIU/L (10 children), such as IgA deficiency (20 vs. 3.7%; Fisher’s exact test, *p* = 0.07; OR = 6.54 [1.14–37.7], *p* = 0.04; φ = 0.18) and total IgG elevation (40 vs. 15.3%; Fisher’s exact test, *p* = 0.07; OR = 3.68 [0.97–13.98], *p* = 0.06; φ = 0.15). In this group, weight deficit was also more frequent at the diagnosis of UC (50 vs. 22.7%, Fisher’s exact test, *p* = 0.06; OR = 3.4 [0.94–12.4], *p* = 1.86; φ = 0.15), while pancolitis was observed less frequently (20 vs. 42%; Fisher’s exact test, *p* = 0.04; OR = 0.15 [0.02–1.22], *p* = 0.07; φ = −0.15). 

Since variables related to sIgE positivity, as mentioned above, correlated with a lower mean age of patients, the presence of parameters such as IgA deficiency, moderate/severe anaemia, pancolitis, fever, IgG elevation and being underweight was analysed for being parallelly correlated with lower age of patients; however, no such phenomenon was noted. Weight loss in the pre-diagnostic period was parallelly correlated with a higher age of patients (mean age 167 months vs. 152; Z = −2.25, *p* = 0.01). IgA deficiency was related to children with a mean age of 142 months, which was not significantly lower than the age of children presenting with normal IgA levels (157 months; Z = 0.92, *p* = 0.18). Furthermore, children with growth impairment were not younger than others (143 vs. 158; Z = 0.7, *p* = 0.23), as well as those with abdominal pain (155 vs. 159 months; *p* > 0.05), fever (150 vs. 158; *p* > 0.05), moderate/severe anaemia (158 vs. 156 months; *p* > 0.05), extensive colitis (55 vs. 156 months; *p* > 0.05) or elevated IgG (147 vs. 159 months; *p* > 0.05).

**Figure 5:**
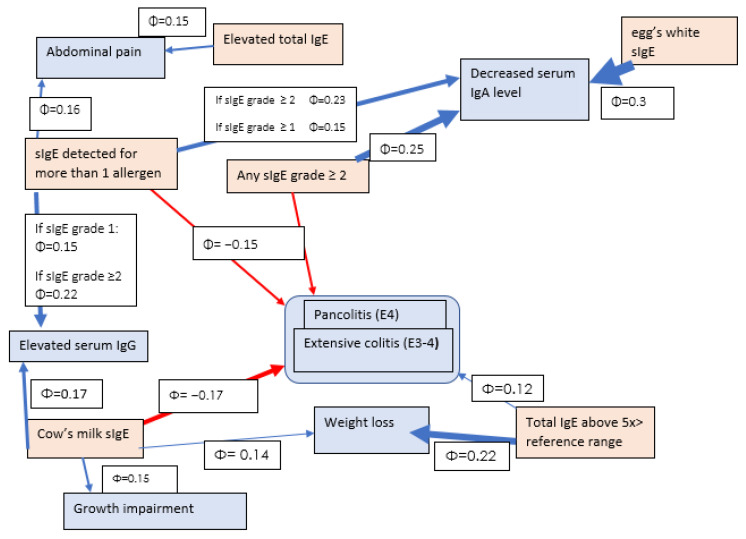
UC correlations.

**Figure 6:**
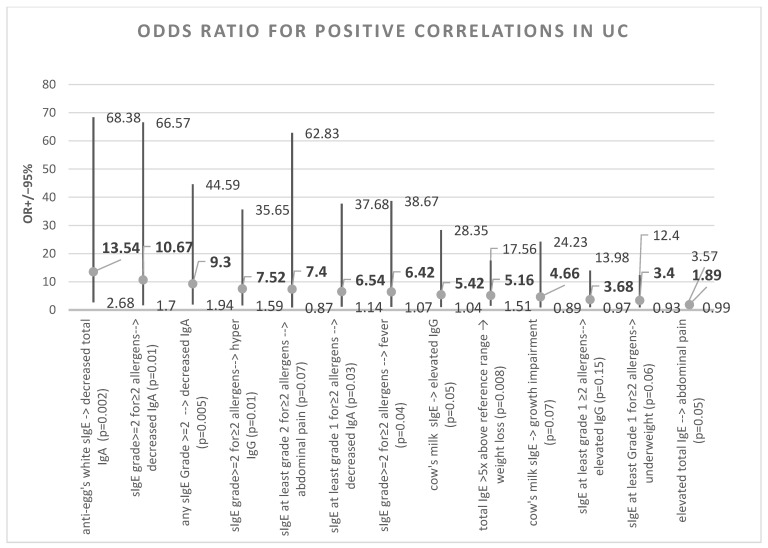
UC—odds ratios for positive correlations.

**Figure 7:**
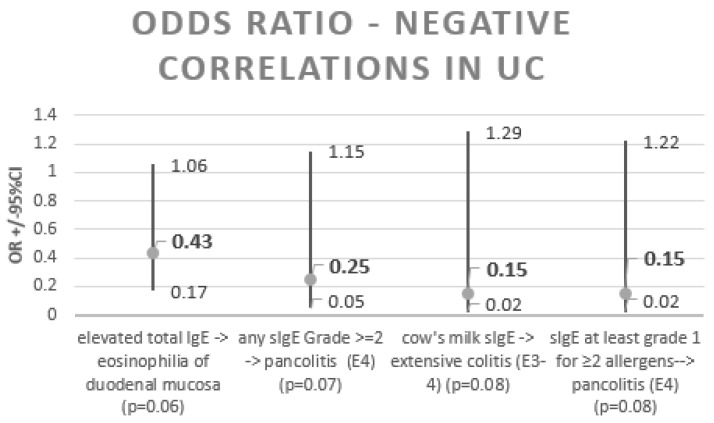
UC—odds ratios for negative correlations.

Correlations, along with their φ value, are shown in Figure 5. Figure 6 and Figure 7 summarise the significant and borderline positive and negative odds ratios for the correlations studied, along with the 95% confidence interval.

Numerous variables were found not to correlate with tested parameters; for example, none of the variables correlated with the risk of biological treatment or the presence of intestinal mucosal eosinophilia. Table S2 in Supplementary Materials summarises all tested correlations, both those statistically significant and non-significant.

## 4. Discussion

According to the European Academy of Allergology and Clinical Immunology (EAACI), 30% of Europeans currently have allergy symptoms [29]. The prevalence of food allergies depends on the methodology of conducted studies; basically, if they are challenge-proven or self-reported. In Western countries, overall challenge-proven food allergy prevalence is estimated at about 4–10% [30] and at 6–8% in children < 5 years of age. [31]. At the same time, the prevalence of paediatric IBD in the USA was estimated at 33.0/100,000 in 2007 and 77.0/100,000 in 2016 (0.033–0.077%) [32]. In general, most of patients with IBD receive their diagnosis between 20–30 years of life. 

The gold standard for the diagnosis of food allergy remains the double-blind placebo-controlled food challenge (DBPCFC), with single-blind or an unblinded oral food challenge (OFC) more commonly used in practice for diagnostic rather than scientific purposes [33]. Additional tests, usually skin prick tests (SPT) or sIgE, remain ancillary tests. Their positive result indicates sensitisation to the allergen, but is not necessarily indicative of clinical allergy, although data are available on the magnitude of skin reaction and sIgE concentration required for the PPV of the tests to reach >95% [28,33].

Hence, the available literature analyses either the coexistence of food allergies and IBD or the presence of sIgE or SPT markers in IBD without determining allergies—as in our study. Regardless of the methodology, the available analyses focus on the epidemiological aspect, assessing the prevalence of allergic disease/abnormal sIgE results in UC or CD compared to healthy controls without attempting to assess the impact of this presence on the IBD picture. 

In the current study, we have chosen to evaluate only the laboratory exponents of food sensitisation, even though they do not necessarily correspond to clinically overt allergies, in order to eliminate diagnostic ambiguities. We were concerned not only about the incompleteness of retrospective data on the performance and course of the diagnostic elimination and OFC, but also about the difficulty of interpreting food challenges in children with chronic inflammatory bowel disease at the time of recent diagnosis, i.e., usually clinically active inflammation. Furthermore, the aim of the study was not to assess the prevalence of food allergies in patients but to assess whether the presence of IgE-dependent sensitisation to food is associated with the phenotype/severity/location or extent of IBD. It was therefore decided to analyse the presence of sIgE as a potential prognostic factor irrespective of its concentration at a threshold of 0.35 KIU/L, i.e., also low concentrations not meeting the 95% PPV criterion for the diagnosis of clinical allergy. The research hypothesis was that circulating serum IgE antibodies to food antigens are relevant to the picture of inflammatory bowel disease already below the threshold, suggesting an independent diagnosis of clinical food allergy. We considered the research utility of possibly confirming that a patient with two comorbidities (IBD and food allergy) has a potentially worse outcome than a patient with one disease (IBD only) to be questionable. In contrast, the demonstration that circulating sIgEs also influence IBD severity without clinically overt allergies leaves room for possible dietary intervention, for which there would be no indication in a separate analysis of allergy alone. This idea seems to be confirmed by the efficacy of the Crohn’s Disease Exclusion Diet (CDED) independent of the presence or absence of food allergy to the eliminated foods [34].

Despite expectations, our material did not show that the coexistence of sIgEs for cow’s milk, egg or wheat is associated with higher disease activity, as measured by PCDAI/PUCAI. Moreover, for Crohn’s disease, we have shown that elevated tIgE is associated with a lower incidence of complicated disease behaviour. Summarising all observed correlations, in the group of children with Crohn’s disease, more statistically significant phenomena were observed for the analysis of high or very high concentrations of total IgE than for food-specific IgE. This may be more consistent with the humoral immune disorders observed in IBD than specifically with allergic mechanisms. We have shown that an elevated tIgE level is associated with a higher incidence of perianal disease, rectal bleeding, being underweight and having pre-diagnosis weight loss, fever, elevated IgG and the presence of ASCA positivity, as well as a positive family history of IBD.

The literature describes various humoral immune disorders in Crohn’s disease patients. Authors Rai et al. noted, for example, major class immunoglobulin deficiency in 22.7% of patients, more frequently in older patients and those with hypalbuminaemia, and more frequently in CD compared with UC. Elevated levels of IgG and IgA were observed in patients with pouchitis [35]. Song et al. found elevated levels of major class antibodies in Crohn’s disease (25% for IgG and 17% for IgA). These abnormally high levels were in correlation with severe disease activity [23]. Lin et al. found that patients with IBD had higher stool IgA and IgG concentrations and a higher percentage of IgA- and IgG-coated bacteria in faeces compared with healthy controls, which may be part of a dysregulated mucosal immune response to commensal microbiota in IBD [36]. Also, these parameters correlated with the clinical and laboratory exponents of disease activity.

The PIBD-Ahead program—a systematic review of different clinical and laboratory parameters of paediatric IBD assessed as potential risk factors for unfavourable events during the course of IBD—concluded that the presence and titre of ASCA in CD correlates with the risk of disease-related surgery, complicated disease course and the need for more aggressive treatment [37]. In our samples, we showed that high tIgE is associated with positive ASCA antibodies, but we did not show an association with the risk of surgical treatment, which may be due to the small number of IBD patients already needing surgical treatment < 18 years of age.

Regarding the correlation of Crohn’s disease characteristics with the presence of sIgE in our samples, we only showed an association of the presence of egg white sIgE with small bowel involvement distal to the ligament of Treitz (L4b), and with the presence of severe growth impairment which may be pathogenetically directly due to small bowel involvement. In our view, the apparent causal link between these two sIgE effects adds credibility to the results as a non-coincidental correlation.

In summary, elevated IgE in Crohn’s disease appears to be associated with a multidirectional increase in the inflammatory activity of the disease. It cannot be ruled out that it is secondary, rather than primary, to high disease activity and independent of the presence or absence of food allergies. In the pathogenesis of Crohn’s disease, altered intestinal permeability (“leaky gut”) is hypothesized, but it remains unclear if it is an early, primary defect or secondary path mechanism leading to exacerbations; most probably, it is both [38,39,40]. 

In contrast, in a group of children with ulcerative colitis, we showed that the presence of cow’s milk sIgE, or the presence of any of the tested sIgEs (egg, wheat, milk) in general, reduces the risk of extensive UC, while strongly increasing the risk of weight loss before diagnosis. Such an observation raises the question of whether the effect of weight loss with the concomitantly less extensive colorectal disease is a result of independently coexisting allergic enteropathy or, for example, of an elimination diet conducted by the patient alone, perhaps in search of symptom relief prior to obtaining a diagnosis. The negative correlation between markers of food sensitisation in UC and the extent of the disease seems particularly interesting in the context of previous studies showing that it is the Th1/Th2 imbalance, which also occurs in allergic diseases, that plays a special role in UC. At the same time, however, it is in UC that no elimination diet has so far been conclusively shown to be effective [41]. This is even though the Crohn’s disease exclusion diet is known to be effective—due at least in part to the elimination of food additives, including emulsifiers—and that the destruction of intestinal mucus plays an important role in UC [42,43]. 

In ulcerative colitis, we also noted further phenomena concerning humoral immunity already observed in the literature, such as a reduction in total IgA levels in patients with egg white sIgE and sIgE at any concentration for more than one allergen. In the literature, it is a well-recognized fact that primary IgA deficiency is a promoting factor for allergic diseases through several mechanisms [44]. In our study group, eight children had reduced IgA relative to the reference value for their age. The estimated prevalence of primary IgA deficiency worldwide is 1:3000 to 1:150, depending on the population studied. In the case of our patients, all children with an abnormal - decreased IgA concentration test result at the time of IBD diagnosis, including those with IgA level slightly decreased in a single measurement, were classified as IgA-reduced for age. Therefore, it would be incorrect to conclude that all these patients had a primary IgA deficiency. However, it is worth recalling that up to 40% of patients with primary IgA deficiency have the allergic disease as their first clinical manifestation [45]. It is probably the result of high penetration of allergens through the mucosa due to the abnormal function of serum and mucosal IgA and dysregulation in the homeostasis of the intestinal microbiome. This is a mechanism highly analogous to the abnormal response to the intestinal microbiome occurring in IBD and manifested, among other things, by the presence of anti-microbial antigens antibodies. Reduced Th1 and Th17 cells compared with healthy controls are also described in cases of IgA deficiency [46]. The role of Th17 cells in the pathogenesis of inflammatory bowel disease is currently in the spotlight, as IL17 has been shown to have a strongly pro-inflammatory effect in the intestinal mucosa in IBD. At the same time, in the healthy state, IL17 is probably precisely responsible for the function of the mucosal barrier [47,48].

In this extremely complex network of interrelationships between IBD and allergic diseases, which reflects the complicated function of the immune system, the most difficult task seems to be to assess the direction of the interactions taking place and the chronology of the phenomena acting on each other. In this context, epidemiological studies that attempt to describe population-based phenomena in terms of their co-occurrence and typical age of onset are an important perspective.

Most of the available work analyses food allergies as historical data obtained from questionnaires [15,16]. A second group of authors has assessed the prevalence of serum-specific IgE or IgG to food proteins [17,18]. Nevertheless, IgG against food antigens is not an approved diagnostic test in allergology and its production is instead considered a normal phenomenon in response to food [19]. Rarely, authors analyse the prevalence of allergic comorbidities with IBD using other methods such as skin prick test [49], and some combine these methods [14].

Wasielewska at al. published in 2019 the paper analysing the prevalence of allergic diseases in children with IBD compared with a control group based on a questionnaire study [14]. Sensitisation to allergens was determined by information on patch skin test results, spot tests or the presence of specific IgE. The allergic diseases analysed were food allergy, cow milk allergy, allergic rhinitis, asthma and atopic dermatitis. Although the researchers mention the above-highlighted methods of assessing sensitisation to allergens, they are not sufficient to diagnose a specific allergic disease and it is likely that the questionnaire included questions about the child’s history of already diagnosed allergic disease, but we are unable to answer these concerns unequivocally without knowing exactly the questionnaire used.

Comparing the results in our samples, sIgE to any of several food allergens tested was found in 7 of 119 children with CD (6%) and in 13 (i.e., 7.5%) of 173 children with UC. These values are comparable to those cited in Wasielewska et al. for the frequency of sensitisation to food allergens tested by diagnostic tests (5 out of 60, i.e., 8%) and correspond to the population prevalence of allergic diseases in Poland and worldwide. At the same time, Wasielewska et al. reported a significantly higher incidence of a definitive diagnosis of food allergy by history in the study group: 32% of children with IBD (using self-reported questionnaires, which many studies consider to result in overestimation of the diagnosis). The authors conclude that, ultimately, the prevalence of allergic diseases did not differ significantly between the study and control groups (IBD vs. healthy controls). In contrast, a history of cow milk allergy was found to be twice as frequent in CD compared to UC.

Wang et al. analysed sIgE levels, with the addition of IgG levels against food allergens, in a retrospective work comparing 40 patients with ulcerative colitis and 97 patients with Crohn’s disease with 50 healthy controls [18]. The authors concluded that sIgEs occurred at a similar frequency in both the patient and healthy control populations, and that there were no statistically significant differences between total serum IgE concentrations between the study groups. At the same time, however, this study showed a very high prevalence of sIgE positivity: 65.2% in CD and 57.1% in UC, with 73.33% and 37.5% of these patients, respectively, having sIgE present against more than 1 allergen of the 13 allergens tested. The authors did not further seek to answer the question of whether the presence of abnormal total IgE and specific IgE antibodies is associated with differences in the clinical presentation of IBD. In our samples, sIgE was documented in noticeably fewer patients, but the antigens we examined (milk, egg) are generally considered transient allergens (in contrast to, e.g., nuts and seafood). According to ESPGHAN, >75% of children achieve tolerance to cow milk protein by 3 years of age and >90% by 6 years of age [50].

It seems possible that primary allergisation with the presence of sIgE for these allergens activates a pathway of changes in the humoral response, ultimately resulting in immunological phenomena already present after the disappearance of the sIgEs themselves through immunotolerance. The earlier average age of onset of atopic diseases relative to IBD may suggest the involvement of an allergic mechanism in the multifactorial development of IBD in its early phase. In conclusion, the complexity of these phenomena requires further analysis, especially analysis that is not limited to assessing the simultaneous occurrence of allergic diseases in IBD, but that collects reliable data from the patient’s entire health history. Rather than asking whether young adults with IBD have a higher prevalence of allergic diseases than their peers, it seems more interesting to ask whether young adults with IBD had more allergic diseases than their peers when they were young children. Nevertheless, even a precise epidemiological determination of these phenomena will not answer the question of what the cause is and what the effect is in this “vicious circle”, or whether we are, for example, dealing with equivalent effects with the same background, e.g., genetic. Of interest in the context of common mechanisms of allergy and IBD are studies documenting that breastfeeding has a protective effect on the risk of both diseases [51,52].

The ever-increasing knowledge of the intersecting immunological phenomena occurring in both food allergies and IBD, the awareness of the lower average age of onset of allergic diseases compared with inflammatory bowel diseases and the observed parallel increase in the incidence of both groups of diseases worldwide mean that the analysis of co-occurrence and possible overlap of allergic and inflammatory phenomena will remain a focus in IBD.

## Figures and Tables

**Table 1:** UC—age and PUCAI in subpopulations. Statistically significant correlations are presented in bold text.

Study Group Definition (n of Children)	Elevated Total IgE (61)	tIgE > 5× Normal Range (13)	Cow’s Milk sIgE (6)	Egg White sIgE (10)	sIgE ≥ 0.35 KIU/L for Any Tested Allergen (17)	sIgE ≥ 0.7 KIU/L for Any Tested Allergen (13)	sIgE > 0.7 KIU/L for ≥2 Allergens (7)	sIgE > 0.35 KIU/L for ≥2 Allergens (10)
Mean age (months)	156	157	158	156	160	159	159	160
Mean age in the rest of UC population	158	161	106	126	123	121	111	101
*p*	0.43	0.39	**0.01**	**0.01**	**0.003**	**0.008**	**0.006**	**0.005**
Mean PUCAI	39.7	42.7	36.7	38	37.4	40	45	39.5
Mean PUCAI in the rest of UC population	40.1	39.7	40.1	40	40.2	39.9	39.9	40
*p*	0.5	0.32	0.26	0.29	0.42	0.4	0.5	0.2

## Data Availability

Data is contained within the Supplementary Material (CD and UC source data.xlsx).

## Supplementary Materials

The following supporting information can be downloaded at: https://www.mdpi.com/article/10.3390/nu15081804/s1, Table S1: Crohn’s disease all tested parameters; Table S2: Ulcerative colitis all tested parameters; Source data.xlsx.
