# Supplementary material for: IgE-Dependent Food Sensitisation and Its Role in Clinical and Laboratory Presentation of Paediatric Inflammatory Bowel Disease
Source: Nutrients. 2023 Apr 7;15(8):1804. doi: 10.3390/nu15081804 (PMC10145321; doi:10.3390/nu15081804)
Supplement: Supplementary file 1 [file nutrients-15-01804-s001.zip › Table S1 - Crohn's disease all tested parameters.pdf]

Table S1 - Crohn's disease- all tested parameters

	**Elevated serum total IgE (40)**	**tIgE >5x normal range (17)**	**Egg white sIgE (6)**	**Any sIgE present (7)**
underweight (41)	Chi^2=1.72 p=0.19<br>OR=1.69 [0.76-3.73]<br>p=0.19<br>Φ=0.12	Chi^2=5.22 (p=0.02)<br>OR=3.27 [1.14-9.39]<br>p=0.03<br>Φ=0.2	Fisher's exact test<br>p=0.34<br>OR=1.97 [0.38-10.25]<br>p=0.8<br>Φ=0.08	Fisher's exact test p=0.54<br>OR=0.75 [0.14-4.04]<br>p=0.74<br>Φ=-0.04
Growth impairment (29)	Chi^2=0.11 (p=0.7)<br>OR=0.8 [0.3-2.1] p=0.7<br>Φ= -0.03	Fisher's exact test p=0.4<br>OR=1.35 [0.43-4.23]<br>p=0.6<br>Φ=0.05	Fisher's exact test<br>p=0.15<br>OR=3.35 [0.64-17.58]<br>p=0.15<br>Φ=0.14	Fisher's exact test p=0.54<br>OR=1.26 [0.23-6.87]<br>p=0.79<br>Φ=0.02
Severe growth impairment (9)	Fisher's exact test p=0.4<br>OR=0.54 [0.1-2.7] p=0.4<br>Φ=-0.07	Chi^2=0.08 (p=0.7)<br>Fisher's exact test p=0.6<br>OR=0.73 [0.08-6.28]<br>p=0.78<br>Φ= -0.03	Fisher's exact test<br>p=0.07<br>OR=7.57 [1.18-48.72]<br>p=0.03<br>Φ=0.23	Fisher's exact test p=0.43<br>OR=2.17 [0.23-20.27]<br>p=0.5<br>Φ=0.06
PCDAI severe (71)	Chi^2=0.003 (p=0.9)<br>OR=1.02 [0.5-2.2] p=0.95<br>Φ=0.005<br>Q= 0.01 (p=0.95)	Chi^2=1.3 (p=0.25)<br>OR=0.55 [0.19-1.54]<br>p=0.26<br>Φ=0.1<br>Q= -0.29 (p=0.22)	Fisher's exact test<br>p=0.54<br>OR=1.37 [0.24-7.81]<br>p=0.7<br>Φ=0.03	Fisher's exact test p=0.59<br>OR=0.9 [0.19-4.19] p=0.88<br>Φ=-0.01
overweight (7)	Chi^2=0.08 (p=0.8)<br>Fisher's exact test p=0.6<br>OR=0.8 [1.14-4.2] p=0.8<br>Φ= -0.03	Fisher's exact test p=0.67<br>OR=1 [0.11-8.87] p=1<br>Φ= 0	Fisher's exact test<br>p=0.31<br>OR=3.57 [0.36-35.54]<br>p=0.28<br>Φ=0.1	Fisher's exact test p=0.35<br>OR=2.94 [0.3-28.53]<br>p=0.35<br>Φ=0.09
Rectal bleeding (36)	Chi^2=4.1 (p=0.04)<br>OR=2.29 [1-5.17] p=0.04<br>Φ=0.19<br>Q= 0.39 (p=0.03)	Chi^2=1.07 (p=0.3)<br>OR=1.74 [0.6-5] p=0.3<br>Φ=0.1<br>Q=0.27 (p=0.28)	Fisher's exact test p=0.6<br>OR=1.15 [0.2-6.56]<br>p=0.88<br>Φ=0.01	Fisher's exact test p=0.64<br>OR=0.9 [0.17-4.9] p=0.9<br>Φ=0.01
Diarrhea (76)	Chi^2=0.8 (p=0.36)<br>OR=1.46 [0.65-3.3] p=0.9<br>Φ=0.08<br>Q=0.19 (p=0.35)	Chi^2=1.26 (p=0.26)<br>OR=1.96 [0.6-6.45]<br>p=0.27<br>Φ=0.1<br>Q=32 (p=0.23)	Fisher's exact test p=0.3<br>OR=2.89 [0.32-25.57]<br>p=0.34<br>Φ=0.09	Fisher's exact test p=0.22<br>OR=3.51 [0.4-30.22]<br>p=0.25<br>Φ=0.11
Abdominal pain (80)	Chi^2=0.14 (p=0.7)<br>OR=1.17 [0.5-2.9] p=0.7<br>Φ=0.03	Chi^2=0.09 (p=0.77)<br>OR=0.85 [0.29-2.5]<br>p=0.77<br>Φ= -0.03	Fisher's exact test<br>p=0.63<br>OR=0.95 [0.17-5.41]<br>p=0.95<br>Φ= -0.006	Fisher's exact test p=0.4<br>OR=0.61 [0.13-2.89]<br>p=0.54<br>Φ=-0.06
Weight loss (60)	Chi^2=4.3 (p=0.04)<br>OR=2.3 [1.04-4.9] p=0.04<br>Φ=0.19	Chi^2=1.92 (p=0.17)<br>OR=2.1 [0.72-6.13]<br>p=0.17<br>Φ=0.12	Fisher's exact test<br>p=0.64<br>OR=1.04 [0.2-5.35]<br>p=0.96<br>Φ=0.004	Fisher's exact test p=0.23<br>OR=0.39 [0.07-2.11]<br>p=0.28<br>Φ=-0.1
Fever (17)	Chi^2=0.02 (p=0.9)<br>OR=1.07 [0.37-3.2] p=0.9<br>Φ=0.01<br>Q= 0.04 (p=0.9)	Fisher's exact test p=0.06<br>OR=0.14 [0.01-2.4]<br>p=0.17<br>Φ=-0.17	Fisher's exact test p=0.2<br>OR=3.23 [0.54-19.22]<br>p=0.2<br>Φ=0.12	Fisher's exact test p=0.27<br>OR=2.56 [0.46-14.4]<br>p=0.29<br>Φ=0.1

Perianal disease (21)	Chi^2=3.9 (p=0.049)<br>OR=2.58 [0.99-6.7] p=0.05<br>Φ=0.18	Fisher's exact test p=0.36<br>OR=1.52 [0.44-5.23]<br>p=0.5<br>Φ=0.06)	Fisher's exact test p=0.3<br>OR=0.33 [0.01-6.04]<br>p=0.45<br>Φ=-0.1	Fisher's exact test p=0.24<br>OR=0.28 [0.02-5.1] p=039<br>Φ=0.12
Extraintestinal manifestations (18)	Chi^2<0.001 (p=0.7)<br>OR=0.98 [0.3-2.9] p=0.9<br>Φ= -0.003	Fisher's exact test p=0.5<br>OR=1.24 [0.32-4.86]<br>p=0.75<br>Φ=0.03	Fisher's exact test<br>p=0.22<br>OR=3.03 [0.51-17.94]<br>p=0.22<br>Φ=0.12	Fisher's exact test p=0.07<br>OR=4.85 [0.99-23.85]<br>p=0.05<br>Φ=0.19
family history of IBD (8)	Chi^2=3.2 (p=0.07)<br>Fisher's exact test p=0.08<br>OR=3.6 [0.8-15.9] p=0.09<br>Φ=0.16	Fisher's exact test p=0.32<br>OR=2.13 [0.39-11.56]<br>p=0.38<br>Φ=0.08	Fisher's exact test<br>p=0.35<br>OR=3.03 [0.31-29.59]<br>p=0.34<br>Φ=0.09	Fisher's exact test p=0.39<br>OR=2.5 [0.26-23.74]<br>p=0.43<br>Φ=0.08
Anaemia (77)	Chi^2=1.15 (p=0.3)<br>OR=0.6 [0.29-1.44] p=0.28<br>Φ=0.09<br>Q= -0.22 (p=0.27)	Chi^2=0.94 (p=0.33)<br>OR=0.59 [0.2-1.71]<br>p=0.33<br>Φ=0.09<br>Q= -0.25 (p=0.31)	Fisher's exact test<br>p=0.39<br>OR=2.34 [0.26-20.75]<br>p=0.45<br>Φ=0.07	Fisher's exact test p=0.62<br>OR=1.14 [0.21-6.15]<br>p=0.88<br>Φ=0.01
Hiperproteinemia (12)	Fisher's exact test p=0.6<br>OR=0.98 [0.28-3.5] p=0.9<br>Φ= -0.002	Fisher's exact test p=0.23<br>OR=2.21 [0.53-9.28]<br>p=0.27<br>Φ=0.1	Fisher's exact test<br>p=0.48<br>OR=1.85 [0.2-17.34]<br>p=0.59<br>Φ=0.05	Fisher's exact test p=0.15<br>OR=4.08 [0.7-23.8] p=0.12<br>Φ=0.15
Low albumin (24)	Chi^2=0.001 (p=0.97)<br>OR=0.98 [0.38-2.54]<br>p=0.97<br>Φ= -0.003	Fisher's exact test p=0.46<br>OR=1.26 [0.37-4.28]<br>p=0.37<br>Φ=0.03	Fisher's exact test<br>p=0.65<br>OR=0.78 [0.09-7.03]<br>p=0.8<br>Φ= -0.02	Fisher's exact test p=0.57<br>OR=0.65 [0.07-5.63]<br>p=0.69<br>Φ= -0.04
Elevated total IgA (10)	Fisher's exact test p=0.55<br>OR=0.8 [0.2-3.4] p=0.8<br>Φ= -0.02	Fisher's exact test p=0.15<br>OR=2.9 [0.67-12.57]<br>p=0.15<br>Φ=0.14	Fisher's exact test<br>p=0.42<br>OR=2.31 [0.24-21.98]<br>p=0.47<br>Φ=0.07	Fisher's exact test p=0.47<br>OR=1.9 [0.2-17.6] p=0.57<br>Φ=0.05
Elevated IgG (8)	Fisher's exact test p=0.26<br>OR=2.08 [0.49-8.8] p=0.3<br>Φ=0.09	Fisher's exact test p=0.09<br>OR=4.16 [0.89-19.34]<br>p=0.07<br>Φ=0.18	Fisher's exact test<br>p=0.65<br>OR=0.95 [0.05-18.43]<br>p=0.98<br>Φ= -0.06	Fisher's exact test p=0.6<br>OR=0.82 [0.04-15.6] p=0.9<br>Φ= -0.07
IgA ASCA + (55)	Chi^2=0.06 (p=0.8)<br>OR=0.9 [0.42-1.95] p=0.8<br>Φ=0.02<br>Q= -0.05 (p=0.8)	Chi^2=2.61 (p=0.09)<br>OR=2.38 [0.82-6.92]<br>p=0.11<br>Φ=0.15	Fisher's exact test<br>p=0.59<br>OR=1.15 [0.22-5.97]<br>p=0.86<br>Φ= 0.02	Fisher's exact test p=0.84<br>OR=0.85 [0.18-3.98]<br>p=0.84<br>Φ= -0.02
IgG ASCA + (61)	Chi^2=4.55 (p=0.03)<br>OR=2.33 [1.06-5.13]<br>p=0.04<br>Φ=0.19	chi2=10.85, p=<0.001<br>OR=9.13 [1.98-42]<br>p=0.004<br>Φ=0.3	Fisher's exact test<br>p=0.64<br>OR=0.95 [0.18-4.9]<br>p=0.95<br>Φ=0.006	Fisher's exact test p=0.47<br>OR=0.7 [0.15-3.26] p=0.65<br>Φ=0.04
ASCA IgA+, IgG+ (42)	Chi^2=0.13 (p=0.7)<br>OR=1.16 [0.52-2.55] p=0.7<br>Φ=0.03	Chi^2=7.51 (p=0.006)<br>OR=4.2 [1.42-12.3]<br>p=0.009<br>Φ=0.25	Fisher's exact test<br>p=0.64<br>OR=0.91 [0.16-5.2]<br>p=0.92<br>Φ= -0.009	Fisher's exact test p=0.52<br>OR=0.72 [0.13-3.88] p=0.7<br>Φ= -0.04

L1/L3 (98) vs Crohn's colitis	Chi^2=0.98 (p=0.3)<br>OR=0.62 [0.24-1.62] p=0.3<br>Φ= -0.09	Fisher's exact test p=0.61<br>OR=1 [0.26-5.98] p=1<br>Φ= 0	Fisher's exact test p=0.3<br>OR=3.02 [0.16-55.72]<br>p=0.46<br>Φ=0.1	Fisher's exact test p=0.25<br>OR=3.53 [0.19-64.12]<br>p=0.85<br>Φ=0.12
L4a (43)	Chi^2=2.05 (p=0.15)<br>OR=1.77 [0.8-3.87] p=0.15<br>Φ=0.13	Chi^2=2.43 (p=0.12)<br>OR=2.25 [0.8-6.35]<br>p=0.13<br>Φ=0.14	Fisher's exact test<br>p=0.12<br>OR=3.79 [0.67-21.64]<br>p=0.13<br>Φ=0.15	Fisher's exact test p=0.21<br>OR=2.5 [0.53-11.72]<br>p=0.25<br>Φ=0.1
L4b (15)	Chi^2=1.4 (p=0.23)<br>Fisher's exact test p=0.19<br>OR=0.45 [0.08-1.7] p=0.24<br>Φ= -0.01	Fisher's exact test p=0.64<br>OR=0.91 [0.19-4.46]<br>p=0.9<br>Φ= -0.1	Fisher's exact test<br>p=0.03<br>OR=8.42 [1.52-46.47]<br>p=0.01<br>Φ=0.26	Fisher's exact test p=0.22<br>OR=3.05 [0.54-17.34]<br>p=0.2<br>Φ=0.12
L4a+ L4b+ (5)	Fisher's exact test p=0.45<br>OR=0.48 [0.05-4.45]<br>p=0.52<br>Φ= -0.06	Fisher's exact test p=0.54<br>OR=1.53 [0.16-14.59]<br>p=0.7<br>Φ=0.03	Fisher's exact test<br>p=0.23<br>OR=5.45 [0.51-58.16]<br>p=0.16<br>Φ=0.14	Fisher's exact test p=0.27<br>OR=4.5 [0.43-46.74] p=0.2<br>Φ=0.13
B complicated (41)	Chi^2=4.32 (p=0.04)<br>OR=0.4 [0.17-0.96] p=0.04<br>Φ= -0.19	Fisher's exact test p=0.2<br>OR=0.52 [0.16-1.7]<br>p=0.28<br>Φ= -0.1	Fisher's exact test<br>p=0.11<br>OR=3.95 [0.69-22.53]<br>p=0.12<br>Φ=0.15	Fisher's exact test p=0.48<br>OR=1.4 [0.3-6.59] p=0.67<br>Φ=0.04
Duodenal eosinophilic infiltrates (10)	Fisher's exact test p=0.55<br>OR=0.83 [0.2-3.41] p=0.8<br>Φ= -0.02	Fisher's exact test p=0.57<br>OR=0.65 [0.08-5.45]<br>p=0.69<br>Φ= -0.04	Fisher's exact test<br>p=0.42<br>OR=2.31 [0.24-21.98]<br>p=0.47<br>Φ=0.07	Fisher's exact test p=0.47<br>OR=1.9 [0.2-17.63] p=0.57<br>Φ=0.05
Colonic eosinophilic infiltrates (11)	Fisher's exact test p=0.29<br>OR=1.74 [0.5-6.09] p=0.86<br>Φ=0.08	Fisher's exact test p=0.51<br>OR=0.58 [0.07-4.8] p=0.6<br>Φ= -0.05	Fisher's exact test<br>p=0.09<br>OR=5.78 [0.92-35.98]<br>p=0.06<br>Φ=0.19	Fisher's exact test p=0.13<br>OR=4.58 [0.78-27.03]<br>p=0.09<br>Φ=0.17
Biologic therapy <18 y (35)	Chi^2=0.1 (p=0.7)<br>OR=0.87 [0.37-2.02]<br>p=0.74<br>Φ= -0.03	Chi^2=0 (p=1)<br>OR=1 [0.32-3.09] p=1	Fisher's exact test<br>p=0.43<br>OR=0.46 [0.05-4.13]<br>p=0.49<br>Φ= -0.06	Fisher's exact test p=0.66<br>OR=0.96 [0.18-5.19]<br>p=0.96<br>Φ= -0.005
Surgical therapy <18 y (12)	Fisher's exact test p=0.6<br>OR=0.99 [0.28-3.5] p=0.98<br>Φ= -0.002	Fisher's exact test p=0.23<br>OR=2.1 [0.53-9.18]<br>p=0.27<br>Φ=0.1	Fisher's exact test<br>p=0.52<br>OR=0.63 [0.03-11.77]<br>p=0.8<br>Φ= -0.08	Fisher's exact test p=0.47<br>OR=0.54 [0.03-9.96]<br>p=0.68<br>Φ= -0.08
