# Supplementary material for: IgE-Dependent Food Sensitisation and Its Role in Clinical and Laboratory Presentation of Paediatric Inflammatory Bowel Disease
Source: Nutrients. 2023 Apr 7;15(8):1804. doi: 10.3390/nu15081804 (PMC10145321; doi:10.3390/nu15081804)
Supplement: Supplementary file 1 [file nutrients-15-01804-s001.zip › Table S2 - Ulcerative colitis all tested parameters.pdf]

Table S2 – Ulcerative colitis - tested parameters

	**Elevated serum tIgE (61)**	**tIgE> 5 x normal range (11)**	**Cow's milk sIgE (6)**	**Egg white sIgE (10)**	**sIgE≥0.35 KIU/l for any tested allergen (13)**	**sIgE≥0.7 KIU/l for any tested allergen (13)**	**sIgE positive for ≥2 allergens ≥0.7 KIU/l**	**sIgE positive for ≥2 allergens ≥0.35 KIU/l**
Growth impairment (32)	Chi^2=0.63, p=0.43 OR=0.71 [0.3-1.66] p=0.43; Φ=-0.06	Fisher's exact test p=0.65 OR=0.96 [0.2-4.65] p=0.96; Φ=-0.004	Fisher's exact test p=0.08 OR=4.66 [0.89-24.24] p=0.07; Φ=0.15	Fisher's exact test p=0.4 OR=0.46 [0.06-3.79] p=0.47; Φ=-0.06	Fisher's exact test p=0.9 OR=0.92 [0.25-3.4] p=0.89 Φ=-0.007	Fisher's exact test p=0.68 OR=1.32 [0.34-5.12] p=0.68; Φ=0.03	Fisher's exact test p=0.12 OR=3.46 [0.74-16.33] p=0.12; Φ=0.13	Fisher's exact test p=0.28 OR=1.94 [0.47-7.94] p=0.36; Φ=0.07
Severe growth impairment (12)	Fisher's exact test p=0.33 OR=0.59 [0.1-2.27] p=0.45 Φ=-0.06	Fisher's exact test p=0.22 OR=2.73 [0.53-14.02] p=0.23; Φ=0.1	Fisher's exact test p=0.36 OR=2.84 [0.3-26.4] p=0.36; Φ=0.07	Fisher's exact test p=0.52 OR=1.53 [0.17-13.24] p=0.69; Φ=0.03	Fisher's exact test p=0.33 OR=1.95 [0.39-9.73] p=0.42 Φ=0.06	Fisher's exact test p=0.22 OR=2.72 [0.53-14] p=0.23; Φ=0.1	Fisher's exact test p=0.4 OR=2.35 [0.26-21.27] p=0.45; Φ=0.06	Fisher's exact test p=0.15 OR=3.83 [0.72-20.45] p=0.12; Φ=0.13
Obesity (6)	Fisher's exact test p=0.64 OR=0.92 [0.16-5.15] p=0.92 Φ=-0.008	Fisher's exact test p=0.38 OR=2.59 [0.28-23.93] p=0.4 Φ=0.07	Fisher's exact test p=0.8 OR=1.91 [0.1-37.68] p=0.67; Φ=0.04	Fisher's exact test p=0.7 OR=1.15 [0.06-21.9] p=0.1; Φ=0.05	Fisher's exact test p=0.9 OR=0.67 [0.04-12.25] p=0.78; Φ=-0.06	Fisher's exact test p=0.62 OR=0.88 [0.05-16.48] p=0.93; Φ=-0.05	Fisher's exact test p=0.78 OR=1.65 [0.09-32] p=0.74; Φ=0.04	Fisher's exact test p=0.7 OR=1.15 [0.06-21.9] p=0.92; Φ=0.05
Overweight / obesity (21)	Chi^2=0.04 (p=0.84) OR=0.9 [0.36-2.39] p=0.84 Φ=-0.02	Fisher's exact test p=0.49 OR=1.35 [0.28-6.56] p=0.71; Φ=0.03	Fisher's exact test p=0.45 OR=0.52 [0.03-9.64] p=0.66; Φ=-0.07	Fisher's exact test p=0.26 OR=0.31 [0.2-15.58] p=0.43; Φ=-0.09	Fisher's exact test p=0.23 OR=0.18 [0.01-3.1] p=0.23; Φ=-0.12	Fisher's exact test p=0.17 OR=0.24 [0.01-4.19] p=0.33; Φ=-0.1	Fisher's exact test p=0.4 OR=0.45 [0.03-8.19] p=0.59; Φ=-0.08	Fisher's exact test p=0.26 OR=0.32 [0.02-5.58] p=0.43; Φ=-0.09
Underweight (42)	Chi^2=0.09, p=0.76 OR=0.89 [0.43-1.86] p=0.76; Φ=-0.02	Fisher's exact test p=0.18 OR=2.08 [0.64-6.74] p=0.22; Φ=0.1	Fisher's exact test p=0.45 OR=1.59 [0.98-8.99] p=0.6; Φ=0.04	Fisher's exact test p=0.55 OR=0.77 [0.16-3.77] p=0.75; Φ=-0.03	Chi2=0.27, p=0.6 OR=1.34 [0.44-4.1] p=0.6; Φ=0.04	Fisher's exact test p=0.39 OR=1.43 [0.42-4.9] p=0.57; Φ=0.04	Fisher's exact test p=0.23 OR=2.44 [0.52-11.38] p=0.26; Φ=0.09	Fisher's exact test p=0.06 OR=3.4 [0.93-12.4] p=0.06; Φ=0.15
Diarrhea (25)	Chi^2=0.29, p=0.59 OR=1.27 [0.53-3.02] p=0.59; Φ=0.04	Fisher's exact test p=0.59 OR=1.08 [0.23-5.2] p=0.92; Φ=0.008	Fisher's exact test p=0.2 OR=3.13 [0.54-18.08] p=0.2; Φ=0.1	Fisher's exact test p=0.56 OR=0.64 [0.08-5.31] p=0.68; Φ=-0.03	Chi2=0.27, p=0.6 OR=1.34 [0.44-4.1] p=0.6; Φ=0.04	Fisher's exact test p=0.59 OR=1.08 [0.23-5.2] p=0.9; Φ=0.01	Fisher's exact test p=0.27 OR=2.49 [0.46-13.59] p=0.29; Φ=0.08	Fisher's exact test p=0.44 OR=1.52 [0.3-7.62] p=0.6; Φ=0.04

Rectal bleeding (61)	Chi^2=1.29, p=0.18 OR=1.76 [0.66-4.69] p=0.26; Φ=0.09	Fisher's exact test p=0.56 OR=0.88 [0.17-4.23] p=0.87; Φ=-0.01	Fisher's exact test p=0.4 OR=2.22 [0.12-40.67] p=0.59; Φ=0.08	Fisher's exact test p=0.22 OR=3.69 [0.2-65] p=0.37; Φ=0.1	Fisher's exact test p=0.13 OR=1.34 [0.44-4.1] p=0.6; Φ=0.13	Fisher's exact test p=0.13 OR=6.47 [0.38-111.22] p=0.2; Φ=0.11	Fisher's exact test p=0.66 OR=0.97 [0.11-8.39] p=0.97; Φ=-0.002	Fisher's exact test p=0.22 OR=3.69 [0.2-65] p=0.37; Φ=0.1
Abdominal pain (79)	Chi^2=3.88, p=0.049 OR=1.89 [>0.99-3.57] p=0.05 Φ=0.15 Q=0.3 (p=0.04)	Chi^2=0.73, p=0.39 OR=1.67 [0.5-5.49] p=0.4; Φ=0.07	Fisher's exact test p=0.28 OR=2.37 [0.42-13.32] p=0.33; Φ=0.08	Fisher's exact test p=0.12 OR=2.85 [0.7-11.43] p=0.14; Φ=0.12	Chi2=0.32, p=0.57 OR=1.33 [0.49-3.64] p=0.57; Φ=0.05	Chi2=0.3, p=0.57 OR=1.38 [0.44-4.28] p=0.58; Φ=0.04	Fisher's exact test p=0.03 OR=7.4 [0.87-62.84] p=0.07; Φ=0.16	Fisher's exact test p=0.29 OR=1.79 [0.49-6.58] p=0.38; Φ=0.07
Weight loss (57)	Chi^2=1.54 p=0.21 OR=1.51 [0.74-2.92] p=0.22; Φ=0.1	Fisher's exact test p=0.007 OR=5.16 [1.51-17.56] p=0.009; Φ=0.22	Fisher's exact test p=0.1 OR=4.23 [0.75-23.8] p=0.1; Φ=0.14	Fisher's exact test p=0.2 OR=2.1 [0.58-7.56] p=0.26; Φ=0.08	Chi2=0.52, p=0.47 OR=1.46 [0.52-4.05] p=0.47; Φ=0.06	Fisher's exact test p=0.45 OR=1.27 [0.4-4.09] p=0.68; Φ=0.03	Fisher's exact test p=0.17 OR=2.79 [0.6-12.93] p=0.19; Φ=0.1	Fisher's exact test p=0.25 OR=2.1 [0.28-7.56] p=0.26; Φ=0.09
Fever (14)	Fisher's exact test p=0.58 OR=1.04 [0.33-3.25] p=0.95; Φ=0.005	Fisher's exact test p=0.68 OR=0.94 [0.11-7.78] p=0.95; Φ=-0.005	Fisher's exact test p=0.65 OR=0.96 [0.05-18.29] p=0.98; Φ=-0.05	Fisher's exact test p=0.54 OR=1.44 [0.17-12.44] p=0.74; Φ=0.03	Fisher's exact test p=0.9 OR=0.73 [0.09-6] p=0.8; Φ= -0.03	Fisher's exact test p=0.65 OR=1.02 [0.12-8.6] p=0.98; Φ=0.002	Fisher's exact test p=0.08 OR=6.42 [1.07-38.67] p=0.04; Φ=0.18	Fisher's exact test p=0.54 OR=1.44 [0.17-12.44] p=0.74; Φ=0.03
Fatigue (27)	Chi^2=1.13, p=0.29 OR=1.57 [0.68-3.6] p=0.29; Φ=0.08	Fisher's exact test p=0.13 OR=2.63 [0.75-9.25] p=0.13; Φ=0.12	Fisher's exact test p=0.65 OR=1.08 [0.12-9.6] p=0.95; Φ=0.005	Fisher's exact test p=0.52 OR=0.58 [0.07-4.79] p=0.61; Φ=-0.04	Fisher's exact test p=0.9 OR=0.69 [0.15-3.22] p=0.64; Φ=-0.04	Fisher's exact test p=0.36 OR=0.43 [0.05-3.42] p=0.42; Φ=-0.06	Fisher's exact test p=0.3 OR=0.34 [0.02-6.05] p=0.46; Φ=-0.09	Fisher's exact test p=0.49 OR=1.37 [0.28-6.83] p=0.7; Φ=0.03
Family history for IBD (9)	Fisher's exact test p=0.32 OR=0.5 [0.1-2.5] p=0.4 Φ=0.07	Fisher's exact test p=0.48 OR=0.59 [0.03-10.64] p=0.72; Φ=0.07	Fisher's exact test p=0.72 OR=1.28 [0.07-24.37] p=0.87; Φ=0.05	Fisher's exact test p=0.58 OR=0.77 [0.04-14.15] p=0.86; Φ=0.06	Fisher's exact test p=0.6 OR=0.44 [0.03-7.9] p=0.58; Φ=-0.08	Fisher's exact test p=0.48 OR=0.59 [0.03-10.64] p=0.72; Φ=0.07	Fisher's exact test p=0.32 OR=3.27 [0.35-30.51] p=0.3; Φ=0.08	Fisher's exact test p=0.44 OR=0.77 [0.04-14.15] p=0.86; Φ=0.06
Severe anaemia (11)	Fisher's exact test p=0.19 OR=0.39 [0.08-1.86] p=0.24 Φ=-0.09	Fisher's exact test p=0.41 OR=0.48 [0.03-8.63] p=0.62; Φ=-0.07	Fisher's exact test p=0.33 OR=3.14 [0.33-29.5] p=0.32; Φ=0.08	Fisher's exact test p=0.49 OR=1.7 [0.2-14.78] p=0.63; Φ=0.04	Fisher's exact test p=0.3 OR=2.18 [0.43-11.02] p=0.35; Φ=0.07	Fisher's exact test p=0.559 OR=1.25 [0.15-10.6] p=0.84; Φ=0.02	Fisher's exact test p=0.63 OR=0.9 [0.05-16.79] p=0.95; Φ=-0.05	Fisher's exact test p=0.49 OR=1.7 [0.2-14.78] p=0.63; Φ=0.03
Hiperproteinemia (25)	Chi^2=2.98, p=0.08 OR=0.41 [0.15-1.16] p=0.09	Fisher's exact test p=0.41	Fisher's exact test p=0.61	Fisher's exact test p=0.56	Fisher's exact test p=0.47	Fisher's exact test p=0.41	Fisher's exact test p=0.67	Fisher's exact test p=0.56

	Φ=-0.13	OR=0.47 [0.06-3.8]<br>p=0.48<br>Φ=-0.06	OR=1.19 [0.13-10.65]<br>p=0.88<br>Φ=0.01	OR=0.64 [0.07-5.31]<br>p=0.68;<br>Φ=-0.03	OR=0.34 [0.04-2.71]<br>p=0.31;<br>Φ=-0.08	OR=0.47 [0.01-3.8]<br>p=0.48;<br>Φ=-0.06	OR=1 [0.11-8.6]<br>p=0.99;<br>Φ<0.001	OR=0.64 [0.08-5.31]<br>p=0.68;<br>Φ=-0.03
PUCAI severe (22)	Chi^2=0.35, p=0.55<br>OR=1.32 [0.53-3.29]<br>p=0.59;<br>Φ=0.05	Fisher's exact test<br>p=0.51<br>OR=1.27 [0.26-6.17]<br>p=0.77;<br>Φ=0.02	Fisher's exact test<br>p=0.44<br>OR=0.5 [0.03-9.14]<br>p=0.64;<br>Φ=-0.07	Fisher's exact test<br>p=0.63<br>OR=0.75 [0.09-6.2]<br>p=0.79;<br>Φ=-0.02	Fisher's exact test<br>p=0.7<br>OR=0.4 [0.05-3.19]<br>p=0.39;<br>Φ=-0.07	Fisher's exact test<br>p=0.49<br>OR=0.55 [0.07-4.46]<br>p=0.58;<br>Φ=-0.04	Fisher's exact test<br>p=0.38<br>OR=0.43 [0.02-7.76]<br>p=0.57;<br>Φ=-0.08	Fisher's exact test<br>p=0.25<br>OR=0.3 [0.02-5.29]<br>p=0.41;<br>Φ=-0.1
Extraintestinal manifestations (37)	Chi^2=1.39, p=0.24<br>OR=0.62 [0.28-1.38]<br>p=0.24;<br>Φ=-0.09	Fisher's exact test<br>p=0.19<br>OR=0.29 [0.04-2.28]<br>p=0.24;<br>Φ=-0.1	Fisher's exact test<br>p=0.38<br>OR=1.89 [0.33-10.72]<br>p=0.47;<br>Φ=0.06	Fisher's exact test<br>p=0.14<br>OR=2.62 [0.7-9.9]<br>p=0.15;<br>Φ=0.11	Fisher's exact test<br>p=0.76<br>OR=1.15 [0.35-3.7]<br>p=0.82;<br>Φ=0.02	Fisher's exact test<br>p=0.58<br>OR=0.64 [0.14-3.07]<br>p=0.59;<br>Φ=-0.04	Fisher's exact test<br>p=0.46<br>OR=1.5 [0.28-8.05]<br>p=0.64;<br>Φ=0.04	Fisher's exact test<br>p=0.64<br>OR=0.91 [0.19-4.5]<br>p=0.91;<br>Φ=-0.01
Extensive disease (E3-4) (97)	Chi^2=0.07, p=0.8<br>OR=1.09 [0.58-2.04]<br>p=0.26;<br>Φ=0.02	Chi^2=2.48, p=0.12<br>Fisher's exact test<br>p=0.1<br>OR=2.8 [0.74-10.55]<br>p=0.13;<br>Φ=0.12	Fisher's exact test<br>p=0.06<br>OR=0.15 [0.02-1.29]<br>p=0.08;<br>Φ=-0.15	Fisher's exact test<br>p=0.53<br>OR=1.19 [0.32-4.37]<br>p=0.8;<br>Φ=0.02	Fisher's exact test<br>p=0.19<br>OR=0.42 [0.13-1.35]<br>p=0.14;<br>Φ=-0.06	Chi2=1.77, p=0.18<br>OR=0.46 [0.15-1.47]<br>p=0.19;<br>Φ=0.1	Fisher's exact test<br>p=0.37<br>OR=0.58 [0.13-2.65]<br>p=0.48;<br>Φ=0.06	Fisher's exact test<br>p=0.08<br>OR=0.32 [0.08-1.26]<br>p=0.1;<br>Φ=-0.13
Backwash ileitis (9)	Fisher's exact test<br>p=0.59<br>OR=0.89 [0.22-3.72]<br>p=0.88;<br>Φ=-0.01	Fisher's exact test<br>p=0.48<br>OR=0.57 [0.03-10.36]<br>p=0.7;<br>Φ=-0.07	Fisher's exact test<br>p=0.76<br>OR=1.47 [0.08-28.77]<br>p=0.8;<br>Φ=0.04	Fisher's exact test<br>p=0.57<br>OR=0.79 [0.04-13.78]<br>p=0.85;<br>Φ=-0.06	Fisher's exact test<br>p=0.6<br>OR=0.43 [0.02-7.74]<br>p=0.57;<br>Φ=-0.08	Fisher's exact test<br>p=0.39<br>OR=0.62 [0.03-11.3]<br>p=0.75;<br>Φ=-0.07	Fisher's exact test<br>p=0.68<br>OR=1.07 [0.06-20.18]<br>p=0.97;<br>Φ=0.05	Fisher's exact test<br>p=0.44<br>OR=0.75 [0.04-13.78]<br>p=0.85;<br>Φ=-0.06
Macroscopic rectal sparing (10)	Fisher's exact test<br>p=0.49<br>OR=0.76 [0.19-3.05]<br>p=0.7;<br>Φ=0.03	Fisher's exact test<br>p=0.44<br>OR=0.51 [0.03-9.25]<br>p=0.65;<br>Φ=0.07	Fisher's exact test<br>p=69<br>OR=1.11 [0.06-21.2]<br>p=0.94<br>Φ=0.05	Fisher's exact test<br>p=0.53<br>OR=0.67 [0.03-12.3]<br>p=0.79;<br>Φ=0.06	Fisher's exact test<br>p=0.6<br>OR=0.38 [0.02-6.86]<br>p=0.52;<br>Φ=-0.08	Fisher's exact test<br>p=0.44<br>OR=0.51 [0.03-9.25]<br>p=0.65;<br>Φ=0.07	Fisher's exact test<br>p=0.65<br>OR=0.97 [0.05-18.02]<br>p=0.98;<br>Φ=0.06	Fisher's exact test<br>p=0.53<br>OR=0.67 [0.04-12.3]<br>p=0.79;<br>Φ=0.06
Any intestinal mucosal eosinophilia (46)	Chi^2=2.45, p=0.12<br>OR=0.55 [0.26-1.17]<br>p=0.12;<br>Φ=-0.12	Fisher's exact test<br>p=0.5<br>OR=0.79 [0.2-3]<br>p=0.73;<br>Φ=-0.03	Fisher's exact test<br>p=0.48<br>OR=0.52 [0.06-4.61]<br>p=0.56<br>Φ=-0.05	Fisher's exact test<br>p=0.27<br>OR=1.86 [0.5-6.9]<br>p=0.36;<br>Φ=0.07	Chi2=0.65, p=0.42<br>OR=1.54 [0.54-4.44]<br>p=0.42;<br>Φ=0.07	Fisher's exact test<br>p=0.49<br>OR=1.2 [0.35-3.13]<br>p=0.77;<br>Φ=0.02	Fisher's exact test<br>p=0.29<br>OR=2.08 [0.45-9.65]<br>p=0.35;<br>Φ=0.07	Fisher's exact test<br>p=0.27<br>OR=1.86 [0.5-6.9]<br>p=0.36;<br>Φ=0.07
Duodenal mucosal eosinophilia (33)	Chi^2=3.5, p=0.06<br>OR=0.43 [0.17-1.06]<br>p=0.06;<br>Φ= -0.14	Fisher's exact test<br>p=0.47<br>OR=1.3 [0.34-5.01]<br>p=0.7;	Fisher's exact test<br>p=0.28<br>OR=0.3 [0.02-5.62]<br>p=0.43;	Fisher's exact test<br>p=0.29<br>OR=1.9 [0.46-7.78]<br>p=0.37;	Chi2=1.3 p=0.25<br>OR=1.9 [0.62-5.8]<br>p=0.26;<br>Φ=0.09	Fisher's exact test<br>p=0.46<br>OR=1.3 [0.34-5.01]<br>p=0.7;	Fisher's exact test<br>p=0.4<br>OR=1.74 [0.32-9.4]<br>p=0.52;	Fisher's exact test<br>p=0.29<br>OR=1.9 [0.46-7.78]<br>p=0.37;

		Φ=0.03	Φ=-0.09	Φ=0.07		Φ=0.03	Φ=0.05	Φ=0.07
Colonic mucosal eosinophilia (17)	Chi^2=0.28, p=0.6 OR=0.74 [0.25-2.22] p=0.6; Φ=-0.04	Fisher's exact test p=0.25 OR=0.3 [0.02-5.34] p=0.42; Φ=-0.1	Fisher's exact test p=0.47 OR=1.89 [0.2-17.17] p=0.57; Φ=0.04	Fisher's exact test p=0.66 OR=1.02 [0.12-8.59] p=0.99; Φ=0.001	Fisher's exact test p=0.68 OR=0.63 [0.07-5.08] p=0.66; Φ=-0.02	Fisher's exact test p=0.37 OR=1.74 [0.36-8.69] p=0.49; Φ=0.05	Fisher's exact test p=0.52 OR=1.56 [0.18-13.8] p=0.69; Φ=0.003	Fisher's exact test p=0.26 OR=2.47 [0.48-12.69] p=0.28; Φ=0.09
IgA deficit (8)	Fisher's exact test p=0.41 OR=0.6 [0.12-3.06] p=0.54; Φ=-0.04	Fisher's exact test p=0.47 OR=1.82 [0.2-16.05] p=0.59; Φ=0.04	Fisher's exact test p=0.25 OR=4.57 [0.47-44.54] p=0.19; Φ=0.1	Fisher's exact test p=0.007 OR=13.54 [2.68-68.38] p=0.002; Φ=0.3	Fisher's exact test p=0.004 OR=11.7 [2.62-52.25] p=0.0013; ϕ=0.3	Fisher's exact test p=0.02 OR=9.3 [1.94-44.6] p=0.005; Φ=0.25	Fisher's exact test p=0.04 OR=10.67 [1.7-66.57] p=0.01; Φ=0.23	Fisher's exact test p=0.07 OR=6.54 [1.14-37.68] p=0.04; Φ=0.18
Elevated IgG (29)	Chi^2=0.9, p=0.34 OR=0.65 [0.27-1.58] p=0.35; Φ=-0.07	Fisher's exact test p=0.32 OR=0.39 [0.04-3.15] p=0.38; Φ=-0.07	Fisher's exact test p=0.06 OR=5.42 [1.03-28.35] p=0.045; Φ=0.17	Fisher's exact test p=0.52 OR=1.26 [0.25-6.26] p=0.78; Φ=0.02	Fisher's exact test p=0.73 OR=1.21 [0.32-4.55] p=0.78; Φ=0.01	Fisher's exact test p=0.62 OR=0.89 [0.19-4.27] p=0.89; Φ=-0.01	Fisher's exact test p=0.02 OR=7.52 [1.59-35.65] p=0.01; Φ=0.22	Fisher's exact test p=0.07 OR=3.68 [0.97-13.98] p=0.06; Φ=0.15
pANCA positivity (21)	Chi^2=0.47, p=0.49 OR=0.7 [0.26-1.9] p=0.5; Φ=-0.05	Fisher's exact test p=0.51 OR=0.58 [0.07-4.73] p=0.61; Φ=-0.04	Fisher's exact test p=0.55 OR=1.47 [0.16-13.23] p=0.34; Φ=0.03	Fisher's exact test p=0.65 OR=0.8 [0.09-6.6] p=0.83; Φ=-0.02	Fisher's exact test p=0.7 OR=0.43 [0.05-3.38] p=0.42; Φ=-0.06	Fisher's exact test p=0.51 OR=0.58 [0.07-4.73] p=0.61; Φ=-0.04	Fisher's exact test p=0.2 OR=3.1 [0.56-17.08] p=0.2; Φ=0.1	Fisher's exact test p=0.35 OR=1.9 [0.37-9.59] p=0.77; Φ=0.06
ASCA IgG (17)	Chi^2=<0.0001, p=0.99 OR=1 [0.35-2.86] p=0.99; Φ<0.01	Fisher's exact test p=0.63 OR=0.75 [0.09-6.15] p=0.79; Φ=-0.02	Fisher's exact test p=0.47 OR=1.89 [0.2-17.17] p=0.57; Φ=0.04	Fisher's exact test p=0.26 OR=2.47 [0.48-12.69] p=0.3; Φ=0.09	Fisher's exact test p=0.22 OR=2.17 [0.56-8.49] p=0.26; Φ=0.09	Fisher's exact test p=0.37 OR=1.76 [0.36-8.69] p=0.49; Φ=0.05	Fisher's exact test p=0.52 OR=1.56 [0.18-13.8] p=0.69; Φ=0.03	Fisher's exact test p=0.26 OR=2.47 [0.48-12.69] p=0.28; Φ=0.09
ASCA IgA (24)	Chi^2=0.5, p=0.48 OR=1.37 [0.57-3.3] p=0.48; Φ=0.05 Q=0.15 (p=0.47)	Fisher's exact test p=0.56 OR=1.14 [0.24-5.5] p=0.16; Φ=0.01	Fisher's exact test p=0.2 OR=3.3 [0.57-19.07] p=0.18; Φ=0.1	Fisher's exact test p=0.15 OR=2.9 [0.7-12.09] p=0.14; Φ=0.12	Fisher's exact test p=0.26 OR=2.09 [0.62-7.05] p=0.23; Φ=0.09	Fisher's exact test p=0.26 OR=1.99 [0.5-7.8] p=0.33; Φ=0.08	Fisher's exact test p=0.25 OR=2.62 [0.48-14.33] p=0.27; Φ=0.09	Fisher's exact test p=0.15 OR=2.89 [0.7-12.09] p=0.14; Φ=0.12
ASCA double positivity (13)	Chi^2=0.06, p=0.8 OR=1.16 [0.36-3.72] p=0.8; Φ=0.02	Fisher's exact test p=0.65 OR=1.03 [0.13-8.59] p=0.03; Φ=0.002	Fisher's exact test p=0.38 OR=2.58 [0.28-23.93] p=0.73; Φ=0.07	Fisher's exact test p=0.17 OR=3.46 [0.65-18.28] p=0.15; Φ=0.12	Fisher's exact test p=0.12 OR=3.13 [0.77-12.71] p=0.11; Φ=0.13	Fisher's exact test p=0.25 OR=2.46 [0.48-12.52] p=0.28; Φ=0.09	Fisher's exact test p=0.43 OR=2.14 [0.24-19.25] p=0.5; Φ=0.05	Fisher's exact test p=0.17 OR=3.46 [0.65-18.28] p=0.15; Φ=0.12
Biological treatment	Chi^2=0.44, p=0.5	Fisher's exact test p=0.57	Fisher's exact test p=0.5	Fisher's exact test p=0.7	Fisher's exact test p=0.7	Fisher's exact test p=0.2	Fisher's exact test p=0.56	Fisher's exact test p=0.7

required (19)	OR=1.39 [0.53-3.65] p=0.5; Φ=0.05	OR=0.66 [0.08-5.36] p=0.7; Φ=-0.03	OR=1.66 [0.18-14.97] p=0.65; Φ=0.04	OR=0.9 [0.1-7.48] p=0.92; Φ=-0.01	OR=0.48 [0.06-3.83] p=0.49; Φ=-0.05	OR=0.27 [0.02-4.7] p=0.37; Φ=-0.1	OR=1.37 [0.16-12.04] p=0.78; Φ=0.02	OR=0.9 [0.1-7.48] p=0.92; Φ=-0.01
